# Enhancement of Catalytic Activity and Durability of Pt Nanoparticle through Strong Chemical Interaction with Electrically Conductive Support of Magnéli Phase Titanium Oxide

**DOI:** 10.3390/nano11040829

**Published:** 2021-03-24

**Authors:** Didem C. Dogan, Jiye Choi, Min Ho Seo, Eunjik Lee, Namgee Jung, Sung-Dae Yim, Tae-Hyun Yang, Gu-Gon Park

**Affiliations:** 1 Fuel Cell Laboratory, Korea Institute of Energy Research (KIER), 152, Gajeong-ro, Yuseong-gu, Daejeon 34129, Korea; didemcil@gmail.com (D.C.D.); jiye1120@kier.re.kr (J.C.); ejlee21@kier.re.kr (E.L.); jimmyim@kier.re.kr (S.-D.Y.); thyang@kier.re.kr (T.-H.Y.); 2 University of Science and Technology (UST), 217, Gajeong-ro, Yuseong-gu, Daejeon 34129, Korea; 3 Graduate School of Energy Science and Technology (GEST), Chungnam National University, 99 Daehak-ro, Yuseong-Gu, Daejeon 34134, Korea; 4 Fuel Cell Research & Demonstration Center, Korea Institute of Energy Research, Buan-gun 56332, Korea; foifrit@kier.re.kr

**Keywords:** polymer electrolyte fuel cells, catalyst support, magnéli phase titanium oxide, size effect, density functional theory

## Abstract

In this study, we address the catalytic performance of variously sized Pt nanoparticles (NPs) (from 1.7 to 2.9 nm) supported on magnéli phase titanium oxide (MPTO, Ti_4_O_7_) along with commercial solid type carbon (VXC-72R) for oxygen reduction reaction (ORR). Key idea is to utilize a robust and electrically conductive MPTO as a support material so that we employed it to improve the catalytic activity and durability through the strong metal-support interaction (SMSI). Furthermore, we increase the specific surface area of MPTO up to 61.6 m^2^ g^−1^ to enhance the SMSI effect between Pt NP and MPTO. After the deposition of a range of Pt NPs on the support materials, we investigate the ORR activity and durability using a rotating disk electrode (RDE) technique in acid media. As a result of accelerated stress test (AST) for 30k cycles, regardless of the Pt particle size, we confirmed that Pt/MPTO samples show a lower electrochemical surface area (ECSA) loss (<20%) than that of Pt/C (~40%). That is explained by the increased dissolution potential and binding energy of Pt on MPTO against to carbon, which is supported by the density functional theory (DFT) calculations. Based on these results, we found that conductive metal oxides could be an alternative as a support material for the long-term fuel cell operation.

## 1. Introduction

Limited supply of fossil fuels and increased emissions of green-house gases will be decisive on world’s future energy crisis. Polymer electrolyte fuel cells (PEFCs) hold great potential as clean energy sources for next-generation electric vehicles with high efficiencies (up to 60%) and zero emissions of green-house gases [1,2,3]. However, remaining challenges such as activity, durability, and cost are preventing the full commercialization of PEFCs [4]. In this regard, the electrocatalyst has been designed to increase the electrochemical active surface area (ECSA) and reduce the amount of platinum (Pt) consumption by alloying with other precious metals (Pd or Ir) and dispersing Pt nanoparticles (NPs) on support materials [5,6]. For that purpose, the porous carbon has been used as a suitable support material with high surface area and electrical conductivity. However, the introduction of a carbon support also brings irreversible electrode degradation under PEFCs operation. Especially, in severe cathode operating conditions, such as low pH, high potentials, and high oxygen concentration, a carbon support is easily corroded. Furthermore, the poor durability of carbon and weak bonding strength with Pt NP causes significant loss of ECSA, oxygen reduction reaction (ORR) activity, and durability.

To alleviate the negative effects of the amorphous carbon deterioration, corrosion-resistant carbon supports; CNT [7,8], CNF [9], and graphene [10,11], heteroatom doped carbon [12] and also metal oxide supports have been introduced in the literature [13,14,15,16,17,18]. Among varied metal oxides [19,20,21], TiO_2_ draws prominent attention because of high chemical-durability and good corrosion-resistance under PEFCs operation. In addition, the hypo d-electron character of TiO_2_ enables its interaction with Pt, known as a strong metal support interaction (SMSI), thus changing the catalytic activity of the noble metal [22,23,24,25]. However, the low electrical conductivity of TiO_2_ results in the increased ohmic resistance of the cell thus limits the application as a support material. For that reason, TiO_2_ is generally mixed with carbon and used as a support for improved durability. On the other hand, some sub-oxides of TiO_2_ (Magnéli phase titanium oxide, MPTO) represent high electrical conductivity which is comparable with graphitized carbon [26]. Considering suitable properties, MPTO is preferred as a support material for several precious metals such as Pt, Ru, and Ir and studied extensively for PEFCs [27].

Previously, Ioroi et al. evaluated the catalytic activity and durability of MPTO. In that paper, Pt/MPTO sample retained their initial ECSA after 10k potential cycles at high potentials (>1.0 V) whereas Pt/VXC-72 sample lost 30–50% of its initial ECSA [28]. They also studied the durability of Pt/Ti_4_O_7_ and Pt/VXC-72 samples by a high-potential holding test (1 h, 1.0–1.5 V). As a result, Pt/Ti_4_O_7_ showed better durability than Pt/VXC-72 sample [29]. Yao et al. showed superior durability as well as nearly 30 times enhanced mass activity (MA) of Pt NPs supported on the fiber-like MPTO in comparison to the commercial Pt/VXC-72 sample [14]. Our group also investigated the activity and durability of Pt NPs on MPTO with a low specific surface area (<1 m^2^ g^−1^). The decrease in the ECSA was observed for Pt/C (94%) after the durability test (0.9–1.3 V, 10k cycles), whereas only 4% of ECSA loss was observed for the Pt/MPTO [30]. Based on the above literature and our finding, it was confirmed that MPTO was suitable support material due to superior resistance to corrosion under high potential conditions. However, few studies have evaluated the durability of Pt/MPTO catalyst in the voltage range (0.6–0.95 V) in which the actual fuel cells are driven. In addition, it is necessary to understand how the interaction between Pt NP and support material affects catalytic performance and durability clearly.

In this work, therefore, the relationship between SMSI and dissolution potential as well as ORR activity were investigated by adopting Pt NPs with varied particle size. To achieve this goal, Pt NP size was systematically controlled to 1.7 nm and 2.9 nm on MPTO and carbon (VXC-72R). In order to maximize the interaction between Pt NP and MPTO, the surface area of the MPTO was further increased by wet milling process. Through the electrochemical studies, we confirmed that the ECSA loss and half-wave potential (E_1/2_) variation of Pt/MPTO samples after the accelerated stress test (AST) 30k cycles are much smaller than that of Pt/C. In addition, it was found that the durability of Pt/MPTO was secured even when the particle size was further reduced from 2.9 nm to 1.7 nm due to the stronger interaction between Pt and support material. The DFT calculations were conducted to explain the behaviors of catalysts with metal oxides-based support material.

## 2. Experimental

### 2.1. Preparation of MPTO

MPTO powder was synthesized by thermal treatment of Rutile TiO_2_ (Sigma-Aldrich, St.Louis, MO, USA) at 1050 °C under H_2_ gas flowing. In order to enhance the specific surface area of MPTO powder, it was further treated by wet milling (NETZSCH, MiniCer, Selb, Germany) process by zirconia beads (0.3 mm, zirconium above 95%, true density 6.05 g cm^−3^, bulk density 3.08 g cm^−3^) with a rotation speed of 4200 rpm. The milling time was set to 100 min to avoid re-oxidation of magnéli phases.

### 2.2. Preparation of Pt/MPTO and Pt/C

Pt NPs of varying sizes (1.7 and 2.9 nm) were deposited on MPTO by the conventional polyol reduction method. Briefly, a necessary amount of MPTO powder was dispersed in H_2_PtCl_6_ dissolved ethylene glycol (EG) solution and then the pH of precursor dispersion was adjusted by the proper amount of 0.1 M NaOH solution. After that, the precursor dispersion was refluxed at 160 °C for 3 h. Through the same procedure, Pt/C (VXC-72) samples were also prepared for comparison. The detail on the synthetic condition is summarized in Table S1. The Pt loadings of the sample were set as 10 wt% for Pt/MPTO and 20 wt% for Pt/C considering the surface area limitations of support materials, respectively.

## 3. Characterizations

### 3.1. Physicochemical Characterizations

Specific surface area of MPTO was determined by using N_2_ adsorption–desorption isotherms at 77 K by the Brunauer–Emmet–Teller (BET) method (ASAP 2020 Micromeritics, Norcross, GA, USA). Before BET measurements, degassing applied at 300 °C for 8 h to remove adsorbed impurities. Pt NP morphology and dispersion were examined by field emission transmission Electron spectroscopy (FE-TEM, Tecnai F20, 200 kV, Hillsboro, OR, USA). The crystal structure of samples was characterized by X-ray diffraction (XRD) measurement (Rigaku RINT2000, Cu Ka, 30 kV, 40 mA, Tokyo, Japan). The Pt loadings on MPTO and carbon were evaluated by inductively coupled plasma–atomic emission spectrometer (ICP-AES, Thermo Scientific iCAP 6500 duo, Waltham, MA, USA).

### 3.2. Electrochemical Characterizations

Rotating-disk electrode (RDE) measurement of samples was recorded with Solartron 1285 potentiostat/galvanostat (Solarton Analytical) in a standard three-electrode cell, with a glassy carbon (GC) disk electrode (projected area 0.196 cm^2^, PINE) coated with thin catalytic film as working electrode, a Pt wire serving as counter electrode, and reversible hydrogen reference electrode (RHE, Gaskatel GmbH, Kassel, Germany) as reference electrode. The electrolyte was prepared with D.I. water and HClO_4_ (ACS reagent 70%) at a concentration of 0.1 M.

Catalyst ink was composed of dispersion with 5 mg of sample powder, 1 mL of D.I. water and isopropyl alcohol (*v*/*v* = 4:1), ethanol solution with a Nafion dispersion (5% *w*/*w*, DuPontTM Nafion^®^). For Pt/MPTO sample, the carbon black was also added for easier observation of well-defined H_UPD_ (underpotential deposition). Then, the catalyst ink was sonicated for 1 h. In advance of the catalyst coating, the GC electrode was cleaned and polished with diamond paste to remove residues. The working electrode was prepared by casting catalyst ink on the GC electrode with a loading of 17 μg_Pt_ cm^−2^ and dried in ambient condition.

Before electrochemical measurements, the catalyst coated GC electrode was cycled between 0.03 V to 1.1 V at 100 mV s^−1^ for 50 cycles to electrochemically clean the surface contamination. The cyclic voltammograms (CVs) of samples were obtained in a potential range of 0.03 V and 1.1 V vs. RHE at a sweep rate of 20 mV s^−1^. ECSA of the samples was calculated by integrating the CV curve for the hydrogen active region and assuming the adsorption of a monolayer of hydrogen on a Pt surface (Q_H_) as 0.210 mC cm^−2^. The ORR activity of the samples was observed by linear sweep voltammograms (LSVs) in an O_2_-saturated 0.1 M HClO_4_ solution with a rotating rate of 1600 rpm at a sweep rate of 10 mV s^−1^. To examine the durability of the samples based on DOE protocol for electrocatalyst, the square wave catalyst AST were performed between 0.6 V (3 s) and 0.95 V (3 s) for 30k cycles [31].

### 3.3. Model Systems and Computational Detail

**Figure 1:**
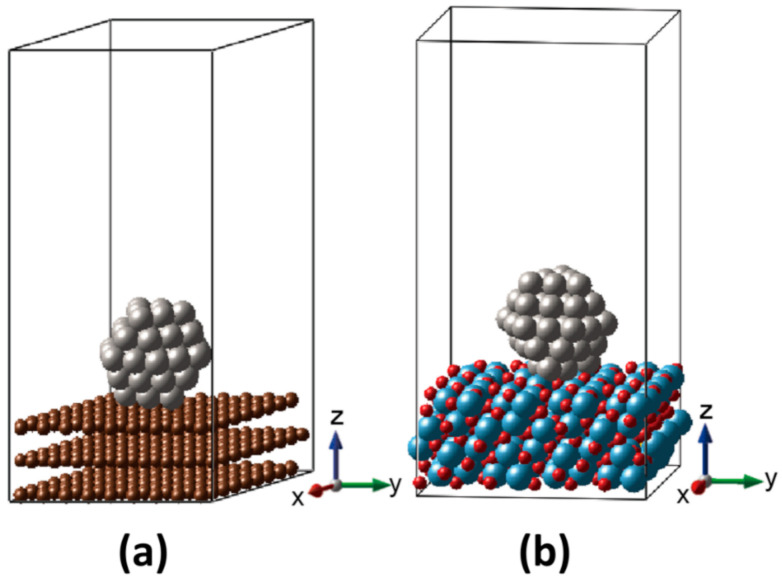
Designed models of (**a**) Pt/C and (**b**) Pt/MPTO.

Pt catalyst was modeled as an NP exposed to vacuum space with and without the supports of MPTO and graphite, as shown in Figure 1. The Pt NPs are composed of 55 Pt atoms sized 1.1 nm as a cuboctahedron enveloped by (111) and (100) facets, and edges in between them. We interfaced the (111) facet with (100) surface of the MPTO support on the ground of thermodynamic surface free energy [32,33,34]_ENREF_1_ENREF_1_ENREF_1._ENREF_1_ENREF_1_ENREF_1 Periodic boundary condition was applied to our model systems with a vacuum space of 29 Å to avoid imaginary interactions. Oxygen adsorption energy was calculated using face-centered cubic (FCC) (111) surfaces of Pt with and without the graphite and MPTO supports.

All calculations were performed using the Vienna ab-initio simulation package [35]. The interactions between ions and electrons were described by Projector Augmented Wave (PAW) pseudo-potential [36,37] implemented in VASP [35]_ENREF_4. The exchange-correlation energy of electrons was described by employing the spin-polarized generalized gradient approximation (GGA) using the Perdew Burke Ernzerhof (PBE) functional [38]. The plane waves were expanded with a cutoff energy of 400 eV until Kohn-Sham equation is converged by the absolute forces within 0.03 eV Å^–1^. Before the slab model of graphite and MPTO were designed, the bulk energies of MPTO and graphite fully relaxed all atoms with a gamma point mesh with 15 × 15 × 15 k-points. The tetrahedron was used method with Blöchl’s corrections [39] in order to calculate the density of states (DOS). The Brillouin zones of Pt/graphene and Pt/MPTO model systems were integrated with a gamma point mesh of 3 × 3 × 1 k-points.

## 4. Results and Discussion

### 4.1. Interaction of Pt NP and Supports

Previous literature reported that the activity and stability of Pt catalyst for ORR in acidic media can be improved by support materials [40,41,42,43]_ENREF_10. Cohesive energy of Pt NP was calculated to understand how chemical interaction with support can enhance electrochemical stability of Pt NP and dispersion of the NPs [42,43]. The Pt binding energy can be defined as Equation (1):

(1) $$E_{BE}^{Pt}=E_{Pt/\mathrm{support}}-E_{Pt}-E_{\mathrm{support}}$$

where $E_{BE}^{Pt}$ is Pt binding energy on graphite and MPTO support. $E_{Pt/\mathrm{support}}$, $E_{Pt}$ and $E_{\mathrm{support}}$ are the total energies of Pt/graphite and Pt/MPTO, the Pt NP, and supports, respectively. Negative binding energy indicates exothermic adsorption of the Pt NP. The binding energies of Pt_55_ particle with the graphite and MPTO were calculated as −0.24 and −11.60 eV, (−0.01 and −0.21 eV/Pt-atom), respectively.

**Figure 2:**
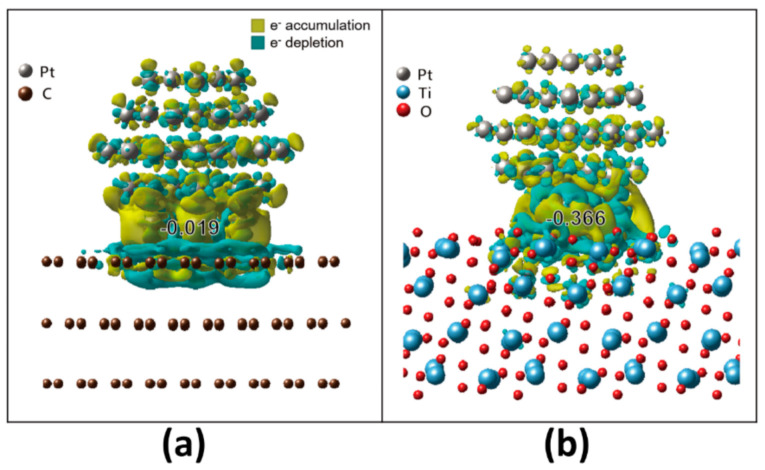
Simulated charge distribution of (**a**) Pt/C and (**b**) Pt/MPTO.

Our results clearly indicate that MPTO is much better for enhancing the Pt NP stability than graphite support. Since the SMSI plays a crucial role in the catalytic activity and stability of Pt NP, we calculated electronic structures of our model systems to elucidate the SMSI mechanism [44,45,46,47,48,49,50], which plays an imperative role in both the catalytic activity and the stability. To understand the origin of the electronic structural change, Bader change analysis [51] was evaluated for Pt/MPTO and Pt/graphite. The differences in the charge density were defined as follows Equation (2):

(2) $$\rho =\rho_{Pt/support}-\rho_{Pt}-\rho_{support}$$

where $\rho_{Pt/support}$, $\rho_{Pt}$ and $\rho_{support}$ are the charge densities of Pt/graphite and Pt/MPTO, Pt NP, and support (e.g., graphite and MPTO), respectively. Figure 2 stands for the charge distribution of Pt/graphite and Pt/MPTO. A negative (positive) *ρ* value represents charge accumulation (depletion). Our DFT calculations show that there are considerable amounts of charge transfer from MPTO support to Pt NP, which negatively polarizes the interface. Furthermore, the charge transfer was much more significant than between graphite and Pt NP. Quantitatively, the Pt atom located at the nearest neighbor of the C and Ti atom gets charges of 0.019 and 0.366 e from the graphite and MPTO, respectively. These contrastingly different electronic interactions render MPTO much better support for Pt NP than the graphite in ORR catalysis.

### 4.2. Ab-Initio Investigation of Durability of Pt NP on Carbon and MPTO

Regarding MPTO as the support, cohesive energy and dissolution potential of Pt NP have been used as the descriptor to understand the electrochemical stability. We calculate the Pt cohesive energy by Equation (3)

(3) $$E_{coh}=-\frac{E_{Pt/support}-E_{support}-nE_{Pt,g}}{n}$$

where $E_{coh}^{}$ is cohesive energy of Pt NP on graphite and MPTO. *E_Pt,g_* is the DFT energy of a single Pt atom in the gas phase, and *n* is the number of Pt atoms. The cohesive energy has been studied as one of the descriptors to identify the stability of the metal NP in the operating condition of fuel cell [46,52,53,54,55]. From Equation (3), the cohesive energy of Pt supported on graphite was 4.86 eV_ENREF_7_ENREF_7, while it is 5.06 eV for MPTO. The graphite does not considerably influence the change of Pt cohesive energy in comparison to the unsupported Pt_55,_ evaluated at (4.85 eV). On the contrary, MPTO significantly enhances the cohesive energy of the Pt NP implying that the stability is enhanced.

The dissolution potential was described as the lowest thermodynamic potential for disintegration followed by dissolution of the outermost shell of Pt NP in an acidic condition [52,54]. To verify that the increased cohesive energy leads to better electrochemical stability, the dissolution potentials were calculated based on the electrochemical dissolution reaction of Equation (4).

(4) $$Pt_{n}Ti_{j}O_{k}C_{l}↔Pt_{n-m}+mPt^{2+}(aq)+2me^{-}+Ti_{j}O_{k}C_{l},$$

where *m* and *n* are the number of Pt atoms in the outermost shell and inner particle, respectively. The *j* and *k* are the total number of *Ti* and *O* in MPTO structure, and *l* is *C* atoms’ number in graphite. The dissolution potential, *U*, can be derived by the Gibbs free energy change from electrochemical reaction, Δ*G* [52,53,54,55]. We assumed a simple dissolution model in which a shell of Pt NP are dissolved to *Pt*^2+^ as reported from our previous investigation [42,52,54,55]. Δ*G* and *U_m_* can be expressed in Equations (5) and (6) [52]_ENREF_20, respectively:

(5) $$\begin{array}{ll} \Delta G= & \mu {}^{\circ}(Pt_{n-m})+m\{\mu {}^{\circ}(Pt^{2+},aq)+kT\ln(a_{Pt^{2+}})\}-2meU_{m}\\ & +\{\mu {}^{\circ}(Ti_{j}O_{k}C_{l},s)+kT\ln(a_{Ti_{j}O_{k}C_{l}})\}\\ & -\{\mu {}^{\circ}(Pt_{n}Ti_{j}O_{k}C_{l},s)+kT\ln(a_{Pt_{n}Ti_{j}O_{k}C_{l}})\} \end{array}$$

(6) $$U_{m}=U_{bulk}+\frac{1}{2me}\{mE(Pt_{bulk})+E(Pt_{n-m})+E(Ti_{j}O_{k}C_{l},s)-E(Pt_{n}Ti_{j}O_{k}C_{l})\}$$

where *μ* is the chemical potentials, *k* is the Boltzmann constant, *a* is the activity coefficient, and *T* is the temperature. *U_m_* and *U_bulk_* are the dissolution potentials of the outermost Pt shell and bulk, respectively. The thermodynamic dissolution potential of bulk Pt is 1.01 V with relative to a standard hydrogen electrode (SHE) for *Pt*^2+^ concentration about 10^−6^ M [55]_ENREF_25. *U_m_* are calculated to 0.62, 0.63 and 0.76 V vs. *SHE* regarding pure Pt particle, Pt/graphite, and Pt/MPTO, respectively as summarized in Table 1. The dramatically enhanced dissolution potential of Pt shows how to improve electrochemical stability for Pt NPs on MPTO support.

### 4.3. Catalytic Properties of MPTO Supported Pt NPs

**Figure 3:**
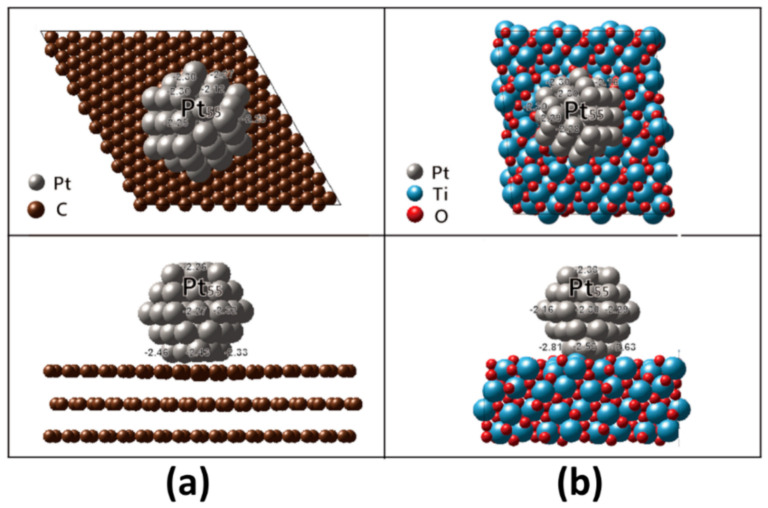
Top and side views of the relaxed Pt_55_ nanoparticle cuboctahedron on (**a**) MPTO (**b**) graphite. The $\varepsilon_{d}$ values are given for each Pt atom in the Pt nanoparticle.

In order to obtain the high catalytic activity, the Sabatier principle should be satisfied, stating that the interactions between catalyst and reactant are neither too strong nor too weak [16]. Therefore, the reactant should have appropriate adsorption strength on a catalyst surface through modulation of the electronic structure of the catalyst [41,56,57,58,59,60]. The d-band center model is a good descriptor in order to understand ORR activity in catalyst surface, according to reports by Nørskov and associates [46,57,58,59,60,61]. The d-band width ($\varepsilon_{d})$ of transition metals is linked to the adsorption strength of the oxygen intermediates [56,61,62]. Even Pt catalyst has demonstrated slow kinetics for ORR due to too strong adsorption energy on the catalyst surface. The association between catalytic activity and oxygen adsorption energy exhibits a volcano plot at which the peak locates at the oxygen adsorption energy positively shifted as much as 0.2 eV from that at the Pt (111) surface [62]. Accordingly, we calculated it for each Pt atom in Pt particle on graphite and MPTO as shown in Figure 3. It has been well known that shifting down d band center of Pt atoms leads to weak oxygen adsorption in Pt skin alloy catalyst [63].

**Figure 4:**
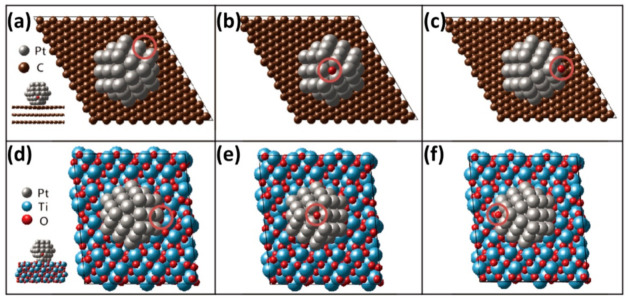
Top view of (**a**–**c**) Pt_55_/graphite and (**d**–**f**) Pt_55_/MPTO where *O* is adsorbed at top, side and bottom on the FCC sites of Pt nanoparticle, as indicated by circles.

As shown in Figure 3, the $\varepsilon_{d}\;$values in Pt atoms nearest supports distribute from −2.33 to −2.46 eV on graphite and from −2.53 to −2.81 eV on MPTO. The $\varepsilon_{d}$ values become more negative on MPTO, guiding to weaker oxygen adsorption energy. By carrying out DFT calculation for oxygen adsorption energy, we evaluated ORR catalytic properties of MPTO and graphite supported Pt NPs. The determination of atomic *O* energy in DFT was derived from H_2_O and H_2_ energies and the oxygen adsorption energy, $E_{ads}^{O}$, on the catalyst surface was calculated as given Equations (7) and (8) [44,64]:

(7) $$\frac{1}{2}E_{O_{2},g}^{}=E_{H_{2}O,l}^{}-E_{H_{2},g}^{}-2.46eV$$

(8) $$E_{ads}^{O}=E_{O-Pt/support}-E_{support}-(\frac{1}{2}E_{O_{2},g}-E_{O-O,\exp})$$

where $E_{O_{2},g}$, $E_{H_{2}O,l}^{}$, and $E_{O-Pt/support}$, are total energies of oxygen, water molecule, and oxygen adsorbed Pt/graphite and Pt/MPTO, respectively. The $E_{O-O,\exp}$ is *O*_2_ bonding energy while experimentally measured −5.23 eV in the literature [65]. Negative (positive) oxygen adsorption energy means exothermic (endothermic) adsorption of an oxygen atom on the Pt surface. Table 1 has summarized the average *O* adsorption energies of top, side and bottom on the FCC sites in Pt_55_ NP deposited on graphite and MPTO (Figure 4). The *O* adsorption energy on unsupported Pt is stronger than that calculated on the FCC site of the bulk Pt (111) surface within (2 × 2) unit cell for a 1/4 *O* monolayer. These results are well consistent that a small Pt NP has slow kinetics for ORR because of stronger *O* bonding on the surface and the influences of the local coordination environment by particle size [66,67,68,69]_ENREF_29_ENREF_35_ENREF_30. The *O* adsorption energies on Pt/MPTO are relatively lower than those of Pt/graphite at the same site due to modification of the Pt electronic structure in which $\varepsilon_{d}\;$is downward shifted. Correspondingly, the results can predict that the ORR activity on MPTO can be improved by these modulated electronic modifications.

### 4.4. Characterization of MPTO and Pt/MPTO

**Figure 5:**
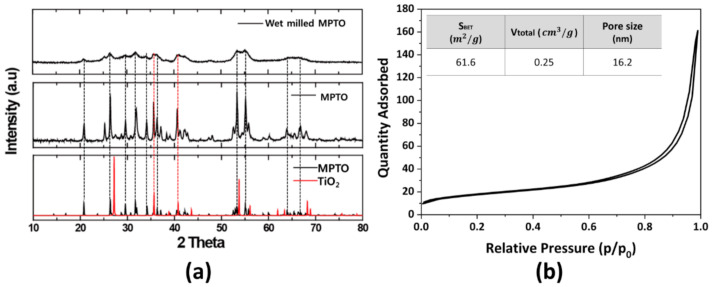
(**a**) X-ray diffraction patterns of wet milled MPTO, as-synthesized MPTO, MPTO and TiO_2_ lattice information obtained from ICSD database. (**b**) N_2_ adsorption/desorption isotherm of wet milled MPTO.

Figure 5a shows the XRD patterns for as-prepared MPTO and wet milled MPTO with crystal structures of TiO_2_ (#169639) and Ti_4_O_7_ (#19017) obtained from inorganic crystal structure database (ICSD). Our calculations depicted at the bottom of Figure 5a indicate that the structures of the TiO_2_ and MPTO are tetragonal and triclinic, respectively. It can be clearly seen that, compared to the peak position of TiO_2_, the as-prepared two mixed phases of TiO_2_ and MPTO is mainly composed of magnèli phases. After wet milling process, MPTO demonstrates broader XRD peaks than as-prepared MPTO due to the formation of smaller particles over the grinding process. According to the BET measurements in Figure 5b, the surface area of as-prepared MPTO increased from 3.7 m^2^ g^−1^ to 61.6 m^2^ g^−1^ after wet milling. In contrast, most of the MPTO samples reported in the literature have a remarkably low specific surface area around 1–2 m^2^ g^−1^, so most of them are limited to a Pt loading amount below 5 wt% [28,29].

After the deposition of Pt NPs on MPTO and carbon, the crystal structure of samples was characterized by XRD patterns in Figure S1. In case of Pt/MPTO samples, the broad three diffraction peaks were observed corresponding to the bulk Pt with face-centered cubic (FCC) structure. Two Pt/C samples showed similar patterns and a broader diffraction peak around 2θ = 40° observed as the particle size decreased due to the inferior crystallinity.

**Figure 6:**
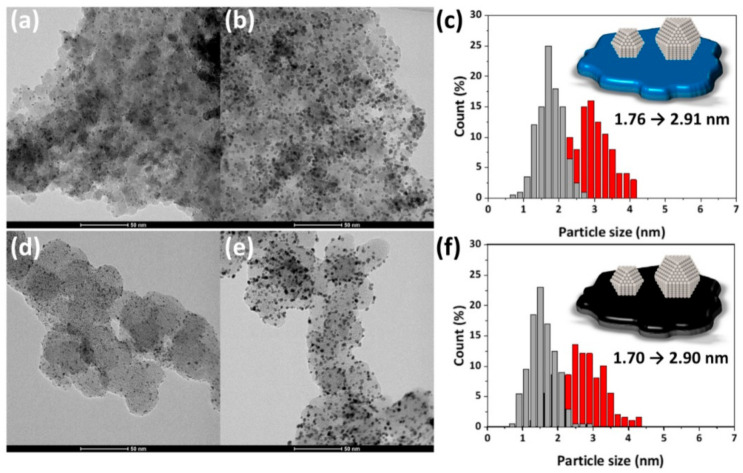
TEM images and particle size histograms of (**a**–**c**) Pt/MPTO and (**d**–**f**) Pt/C.

As shown in Figure 6, the low-magnification TEM images show that Pt/MPTO and Pt/C samples have remarkably high dispersion and uniform distribution of Pt NPs without agglomeration on the corresponding support. From TEM images and particle size histograms, we also found that the particle size was precisely controlled to the desired level (1.7 nm and 2.9 nm) through pH control during the polyol synthesis process. ICP analysis shows that target Pt loadings are successfully achieved for all samples (Table 2)

**Figure 7:**
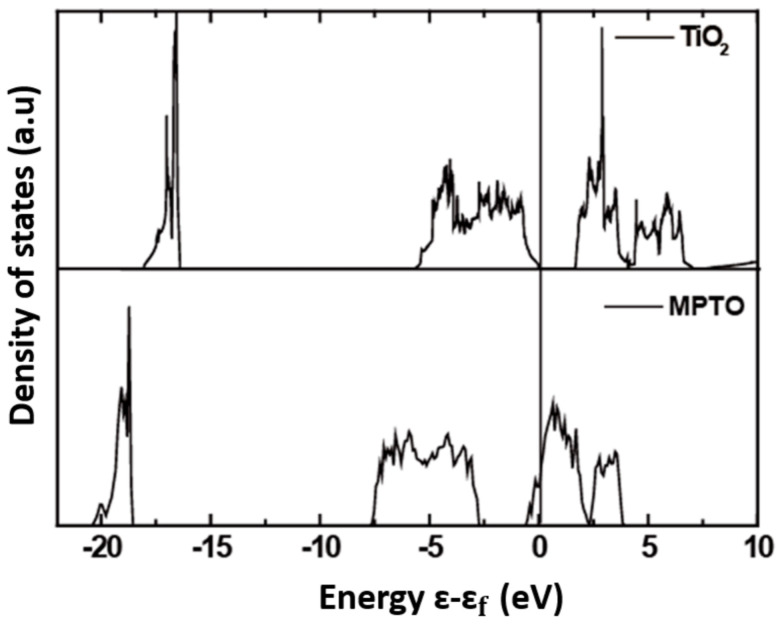
Density of state (DOS) for TiO_2_ (up) and Ti_4_O_7_ (bottom), respectively.

The density of states (DOS) was investigated to obtain an insight from electronic structures of TiO_2_ and MPTO materials as shown in Figure 7. It denotes that DOS of TiO_2_ features poor electrical conductivity as represented by the band gap of ca. 1.69 eV around the Fermi level, well agreeing with the previous literatures [70,71]_ENREF_6. To the contrary, MPTO has energy states for electrons around the Fermi level enabling electrical conductivity, which is critical to be good support material for the Pt catalyst to ORR. Since our experimental sample for MPTO material is composed of two mixed phases of TiO_2_ and MPTO as measured by XRD analysis, the electrical conductivity is lower than pure MPTO.

### 4.5. Electrochemical Studies

**Figure 8:**
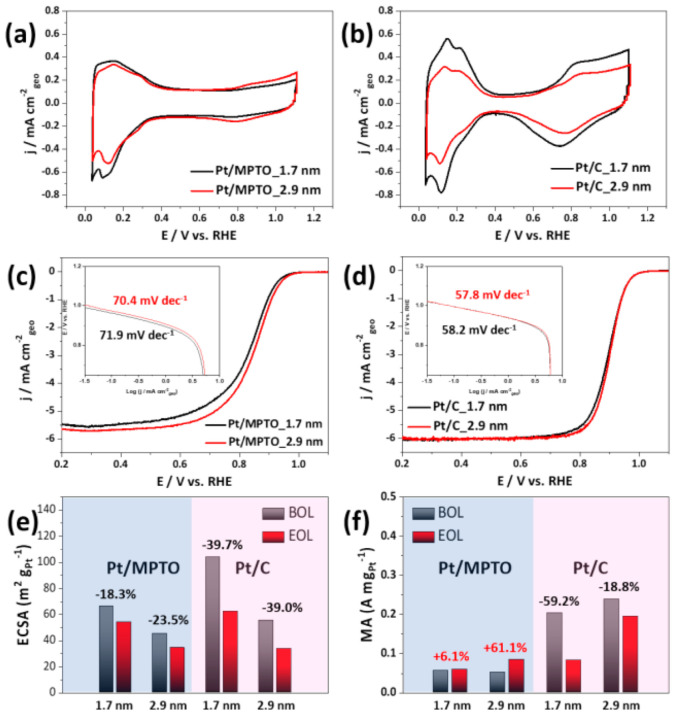
(**a**,**b**) CVs of Pt/MPTO and Pt/C, (**c**,**d**) LSVs, (**e**) ECSAs, (**f**) MA at 0.9V of Pt/MPTO and Pt/C. (BOL: beginning of life, EOL: end of life).

Figure 8a,b presents the CV curves of Pt/MPTO and Pt/C in N_2_-saturated 0.1 M HClO_4_ solution at room temperature. The ECSA of the catalysts was obtained by calculating the charge using the hydrogen adsorption-desorption area. The ECSA of samples are 66.3, 45.1 m^2^ g^−1^_Pt_ for Pt/MPTO samples and 103.7, 55.4 m^2^ g^−1^_Pt_ for Pt/C samples, respectively. The calculated initial ECSA of Pt/MPTO samples is relatively low than Pt/C regardless of particle size. It is due to the low electrical conductivity of MPTO and is consistent with the results reported in previous literature [13,72,73]. The linear sweep voltammograms (LSVs) on Pt/MPTO and Pt/C, recorded in the anodic sweep mode in an O_2_-saturated 0.1 M HClO_4_ solution at 1600 rpm. As shown in Figure 8c,d, the half-wave potential (E_1/2_) of samples is slightly shifted to higher potential as an increase of particle size, indicating the improved ORR activity. As the particle size increases from 1.7 nm to 2.9 nm, the reduced oxophilicity of Pt on MPTO and carbon leads to an increase in ORR performance through the blocking of oxygen species (OH) on active sites [74]. In addition, in the literature, it is well-known that the optimal particle size for ORR is nearly 3.5 nm [75]. Moreover, compared to Pt/C, we confirmed the suppression of Pt-OH formation, which is considered to be due to changes in the electronic structure of Pt by MPTO.

In order to compare the kinetic information of samples for ORR, we calculated the Tafel slope from LSVs as shown in Figure 8c,d. We confirmed that Pt/MPTO samples (70.4–71.9 mV dec^−1^) has a higher Tafel slope value than Pt/C (57.8–58.2 mV dec^−1^), indicating relative slow ORR kinetics. This is expected to be due to the low conductivity of MPTO. In addition, a slight decrease in Tafel slop of Pt/MPTO and Pt/C was observed with an increase in particle size, which is reported to be due to the particle size effect [76]. Consistent with the Tafel slope range (50–80 mV dec^−1^) of Pt reported in the literature, we predict that all samples follow the 4-electron pathway and dissociative mechanism for ORR.

In order to observe the durability of samples, the AST were conducted in N_2_-saturated 0.1 M HClO_4_ at room temperature by square wave potential cycling at 0.6V (3 s) and 0.95 (3 s) V for 30 k cycles. Figures S2 and S3 show the CVs and LSVs curves of Pt/MPTO and Pt/C samples before and after AST 30k cycles. In case of Pt/MPTO samples, one with smaller particle size shows the less ECSA loss (18.3%) compared with larger one (23.5%). In spite of the small Pt particle size with low dissolution potential, its high durability is expected to be due to the strong interaction between Pt and MPTO. On the other hand, Pt/C samples show the larger ECSA loss (39.7, 39.0%) regardless of Pt particle size.

Durability of samples for ORR was also evaluated in Figure S3. Comparison of half-wave potential variation in LSVs curves before and after Pt/MPTO samples, we confirmed that the ORR activity is still remained after AST 30k cycles. In contrary, Pt/C sample with smaller particle size showed the larger drop in half-wave potential by a degradation of Pt NPs due to their low dissolution potential. Interestingly, Pt/C with larger particle size shows almost same LSVs curves before and after AST 30k cycles. We expected that the relatively high dissolution potential and good dispersity of Pt NPs on carbon are the main reason for maintaining ORR performance. As shown in Figure S4, we confirmed the particle size and distribution of Pt/MPTO and Pt/C samples after AST 30k cycles. In case of Pt/C, the particle agglomerate was observed on carbon support due to Ostwald ripening under AST test. On the other hand, Pt/MPTO sample showed similar particle size (~3 nm) and distribution compared with as-prepared Pt/MPTO sample in Figure 6b. Such enhanced durability of Pt/MPTO can be explained due to the strong interaction between Pt and MPTO with higher corrosion resistance. Figure 8f shows the mass activity (MA) of samples at 0.9 V before and after AST 30k cycles. Although the Pt/MPTO samples have relatively low ORR activity than Pt/C, the performance slightly increased after AST 30k cycles. On the other hand, both Pt/C samples have a 59.2, 18.8% of drop in MA, respectively. We also confirm that the Pt/MPTO samples in the literature show a nearly 50% reduction in ECSA and MA after 3k cycles [12]. We expected that the high durability of our Pt/MPTO sample compared to the literature is due to the improved SMSI effect by well-dispersion of Pt NPs on the MPTO with higher surface area. The results are well consistent with DFT prediction which was demonstrated for increasing dissolution potential of Pt nanoparticle on MPTO, indicating an enhanced stability of Pt/MPTO. Based on the electrochemical evaluation, we conclude that MPTO could be alternative to support material for long-term PEFCs operation.

## 5. Conclusions

The motivation of this study is the investigation of catalytic activity and durability by adopting magnèli phase titanium oxide as a support material for PEFCs. For that, MPTO was synthesized and a wet milling process was conducted to obtain the proper surface area for enhancement of the SMSI effect. In the case of a bulk structure, the preparation of perfect MPTO is possible, but in MPTO manufactured to secure surface area, the presence of partially formed TiO_2_ decreases electrical conductivity, and as a result, it was the main reason of understate of ORR activity. Through the results of AST for 30k cycles, superior durability at the voltage cycling (0.6–0.95 V) environment was observed for the MPTO despite mixed phases compared to the carbon regardless of Pt particles size. The excellent durability of Pt/MPTO could be attributed to the higher dissolution potential, cohesive energy, and binding energy that comes from MPTO support, these are explained by the DFT calculations. Based on this synergetic approach of combining experimental and computational investigations, we demonstrated that conductive metal oxide with the appropriate surface area could be promising support material for long-term PEFCs operation.

## Figures and Tables

**Table 1:** The oxygen adsorption strengths, cohesive energies, and dissolution potentials regarding Pt bulk, a Pt nanoparticle, Pt/graphite and Pt/MPTO.

Atomic Oxygen (eV/O)								
	Pt (111) ^1^	Pt_55_ NP	Pt_55_/Graphite			Pt_55_/MPTO		
	Pt_-FCC_	Pt_-FCC_	top_-FCC_	side_-FCC_	bottom_-FCC_	top_-FCC_	side_-FCC_	bottom_-FCC_
E_ads_, eV	−3.92	−4.39	−3.79	−3.59	−3.75	−3.57	−3.62	−3.74
ΔE_O_−ΔE_O_^Pt(111)^, eV	0	−0.47	0.12	0.32	0.16	0.34	0.30	0.17
E_coh_, eV		4.85	4.86			5.06		
U_diss-Pt shell_ vs. SHE, eV	-	0.62	0.63			0.76		

^1^ Oxygen adsorption strength for a 1/4 monolayer of O on a (2 × 2) unit cell of Pt slab model.

**Table 2:** Pt loading, TEM particle size and XRD particle size data.

Samples	Pt Loading (ICP-MS, wt%)	Particle Size (TEM, nm)	Particle Size (XRD, nm)
Pt/MPTO	11.4	1.76	-
Pt/MPTO	8.6	2.91	-
Pt/C	19.2	1.70	-
Pt/C	20.1	2.90	3.00

## Data Availability

Not applicable.

## Supplementary Materials

The following are available online at https://www.mdpi.com/2079-4991/11/4/829/s1, Figure S1: X-ray diffraction patterns of Pt/MPTO and Pt/C, Figure S2: CVs of (a) Pt/MPTO_1.7 nm, (b) Pt/MPTO_2.9 nm and (c) Pt/C_1.7 nm and (d) Pt/C_2.9 nm before and after AST 30k cycles, Figure S3: LSVs of (a) Pt/MPTO_1.7 nm, (b) Pt/MPTO_2.9 nm and (c) Pt/C_1.7 nm and (d) Pt/C_2.9 nm before and after AST 30k cycles, Table S1: Synthetic condition for Pt/MPTO and Pt/C samples
