# Supplementary material for: Enhancement of Catalytic Activity and Durability of Pt Nanoparticle through Strong Chemical Interaction with Electrically Conductive Support of Magnéli Phase Titanium Oxide
Source: Nanomaterials (Basel). 2021 Mar 24;11(4):829. doi: 10.3390/nano11040829 (PMC8063942; doi:10.3390/nano11040829)
Supplement: Supplementary file 1 [file nanomaterials-11-00829-s001.pdf]

Enhancement of Catalytic Activity and Durability of Pt Nanoparticle through Strong Chemical Interaction with Electrically Conductive Support of Magnéli Phase Titanium Oxide

Didem C. Dogan [1,3,†], Jiye Choi [1,4,†], Min Ho Seo [2,†] Eunjik Lee [1], Namgee Jung [4,*], Sung-Dae Yim [1], Tae-Hyun Yang [1] and Gu-Gon Park [1,3,*]

[1] Fuel Cell Laboratory, Korea Institute of Energy Research (KIER), 152, Gajeong-ro, Yuseong-gu, Daejeon 34129, Korea; didemcil@gmail.com (D.C.D.); jiye1120@kier.re.kr (J.C.); ejlee21@kier.re.kr (E.L.); jimmyim@kier.re.kr (S.-D.Y.); thyang@kier.re.kr (T.-H.Y.)

[2] Fuel Cell Research & Demonstration Center, Korea Institute of Energy Research, Buan-gun 56332, Korea; foifrit@kier.re.kr

[3] University of Science and Technology (UST), 217, Gajeong-ro, Yuseong-gu, Daejeon 34129, Korea

[4] Graduate School of Energy Science and Technology (GEST), Chungnam National University, 99 Daehak-ro, Yuseong-Gu, Daejeon 34134, Korea

* Correspondence: njung@cnu.ac.kr (N.J.); gugon@kier.re.kr (G.-G.P)

† These authors have contributed equally to this work and share first authorship

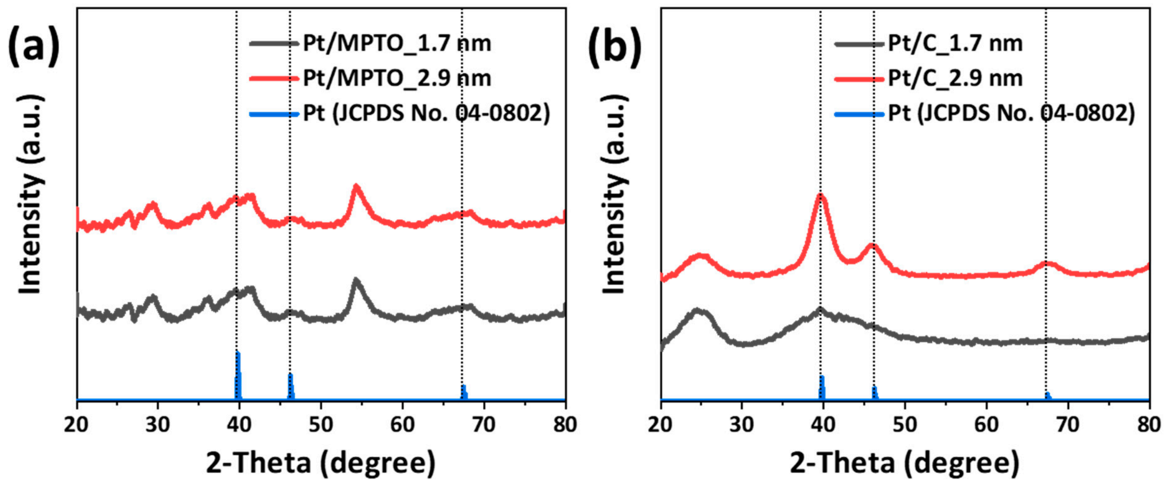

Figure S1. X-ray diffraction patterns of Pt/MPTO and Pt/C.

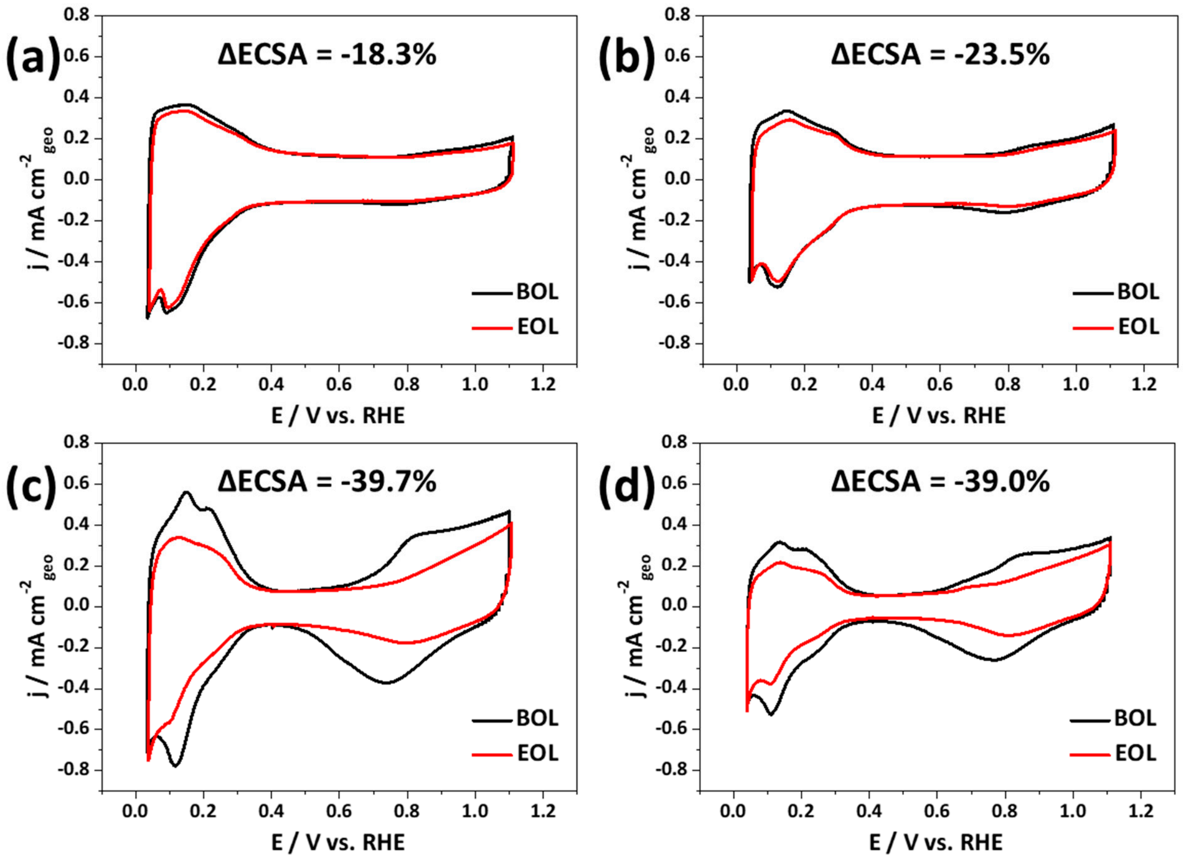

Figure S2. CVs of (a) Pt/MPTO_1.7 nm, (b) Pt/MPTO_2.9 nm and (c) Pt/C_1.7 nm and (d) Pt/C_2.9 nm before and after AST 30k

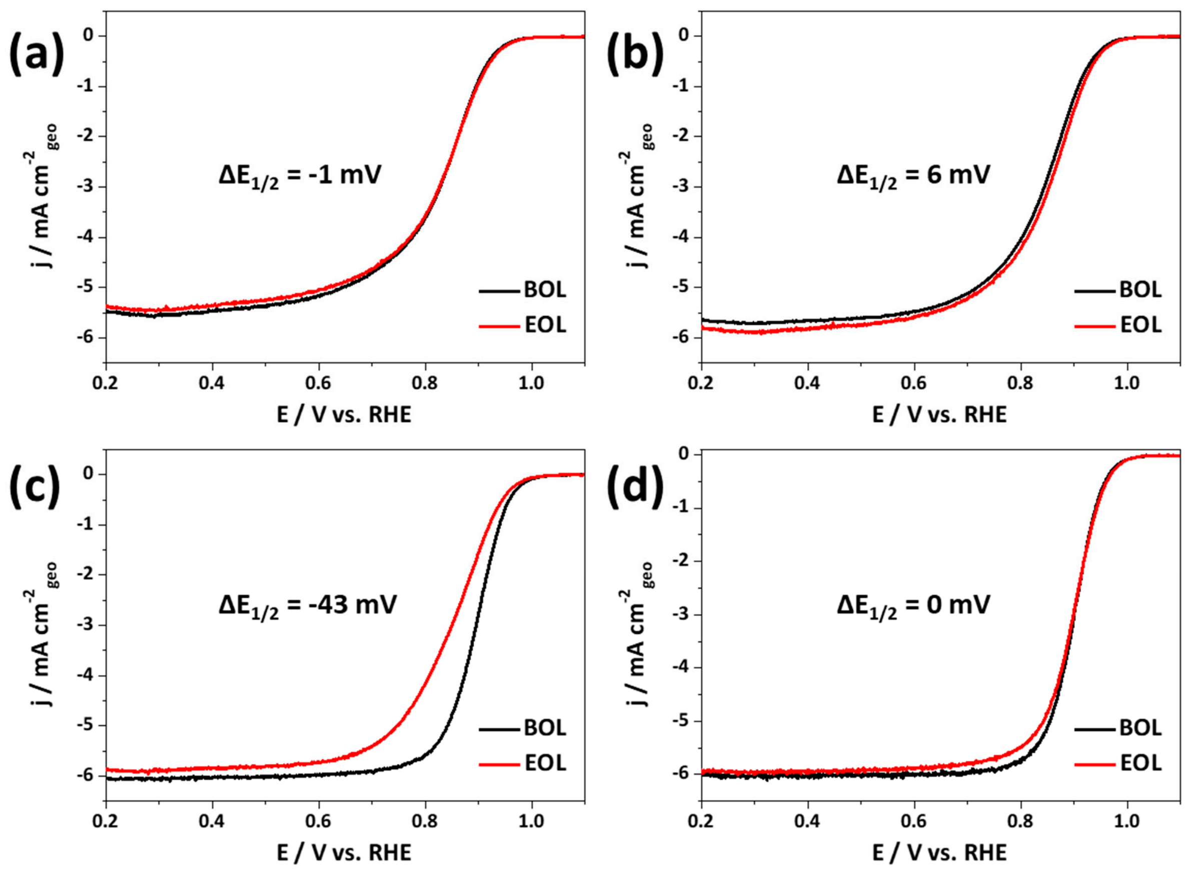

Figure S3. LSVs of (a) Pt/MPTO_1.7 nm, (b) Pt/MPTO_2.9 nm and (c) Pt/C_1.7 nm and (d) Pt/C_2.9 nm before and after AST 30k

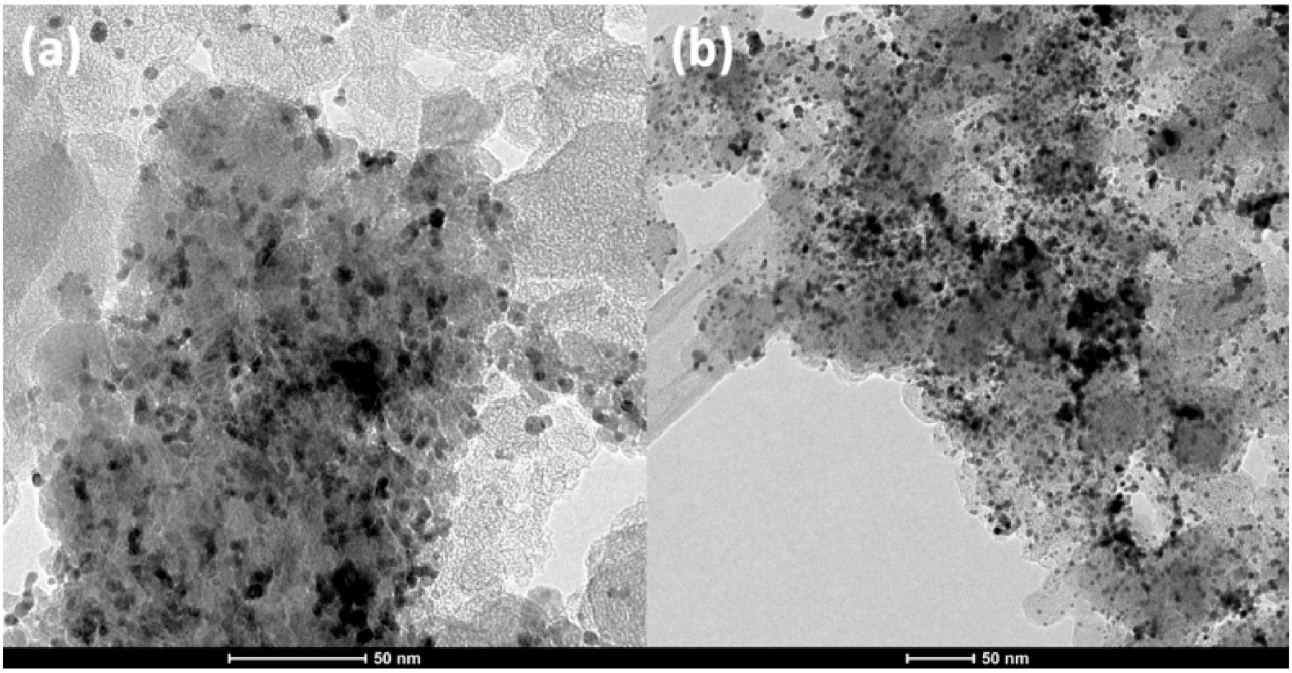

Figure S4. TEM images of (a) Pt/MPTO_2.9 nm and (b) Pt/C _2.9 nm after AST 30k

Table S1. Synthetic condition for Pt/MPTO and Pt/C samples

**Sample**	**Batch (g)**	**Volume of ethylene glycol (mL)**	**Volume of Pt Precursor (mL)***	**Volume of 0.075M NaOH (mL)**	**Target metal loading (wt%)**
Pt/MPTO_1.7 nm	0.1	800	0.1	4.0	10
Pt/MPTO_2.9 nm	0.1	800	0.1	7.0	10
Pt/C_1.7 nm	0.1	800	0.2	18	20
Pt/C_2.9 nm	0.1	800	0.2	10	20

* The concentration of Pt precursor is 1.0 g per 10 mL.
